# A review of flash glucose monitoring in type 2 diabetes

**DOI:** 10.1186/s13098-021-00654-3

**Published:** 2021-04-09

**Authors:** Marcio Krakauer, Jose Fernando Botero, Fernando J. Lavalle-González, Adrian Proietti, Douglas Eugenio Barbieri

**Affiliations:** 1 Department of Technology (Coordinator) SBD–Brazilian Diabetes Society, Science Valley Research Center, Liga de Diabetes, ABC Medical School, Avenida 12 de Outubro 286, Santo André, SP CEP 09030-640 Brazil; 2 Clínica Integral de Diabetes-CLID, Medellín, Colombia; 3 Servicio de Endocrinología, Hospital Universitario Dr José e Gonzalez, UANL, Monterrey, México; 4 Institute of Diabetes and Applied Technology (IDTA), Buenos Aires, Argentina; 5 Abbott Diabetes Care, Sao Paulo, Brazil

**Keywords:** Flash glucose sensing, Intermittent-scanned continuous interstitial glucose monitoring, Type 2 diabetes

## Abstract

**Background:**

Continuous glucose monitoring systems are increasingly being adopted as an alternative to self-monitoring of blood glucose (SMBG) by persons with diabetes mellitus receiving insulin therapy.

**Main body:**

The FreeStyle Libre flash glucose monitoring system (Abbott Diabetes Care, Witney, United Kingdom) consists of a factory-calibrated sensor worn on the back of the arm which measures glucose levels in the interstitial fluid every minute and stores the reading automatically every 15 min. Swiping the reader device over the sensor retrieves stored data and displays current interstitial glucose levels, a glucose trend arrow, and a graph of glucose readings over the preceding 8 h. In patients with type 2 diabetes (T2D) receiving insulin therapy, pivotal efficacy data were provided by the 6-month REPLACE randomized controlled trial (RCT) and 6-month extension study. Compared to SMBG, the flash system significantly reduced the time spent in hypoglycemia and frequency of hypoglycemic events, although no significant change was observed in glycosylated hemoglobin (HbA1c) levels. Subsequent RCTs and real-world chart review studies have since shown that flash glucose monitoring significantly reduces HbA1c from baseline. Real-world studies in both type 1 diabetes or T2D populations also showed that flash glucose monitoring improved glycemic control. Higher (versus lower) scanning frequency was associated with significantly greater reductions in HbA1c and significant improvements in other measures such as time spent in hypoglycemia, time spent in hyperglycemia, and time in range. Additional benefits associated with flash glucose monitoring versus SMBG include reductions in acute diabetes events, all-cause hospitalizations and hospitalized ketoacidosis episodes; improved well-being and decreased disease burden; and greater treatment satisfaction.

**Conclusion:**

T2D patients who use flash glucose monitoring might expect to achieve significant improvement in HbA1c and glycemic parameters and several associated benefits.

## Background

Self-monitoring of blood glucose (SMBG) is a well-established approach for daily management of glycemic control in persons with diabetes mellitus, including those with type 2 diabetes (T2D) [1, 2]. In recent years, continuous glucose monitoring (CGM) systems have increasingly been adopted as an alternative or adjunct to SMBG by patients receiving insulin therapy [3, 4].

**Fig. 1:**
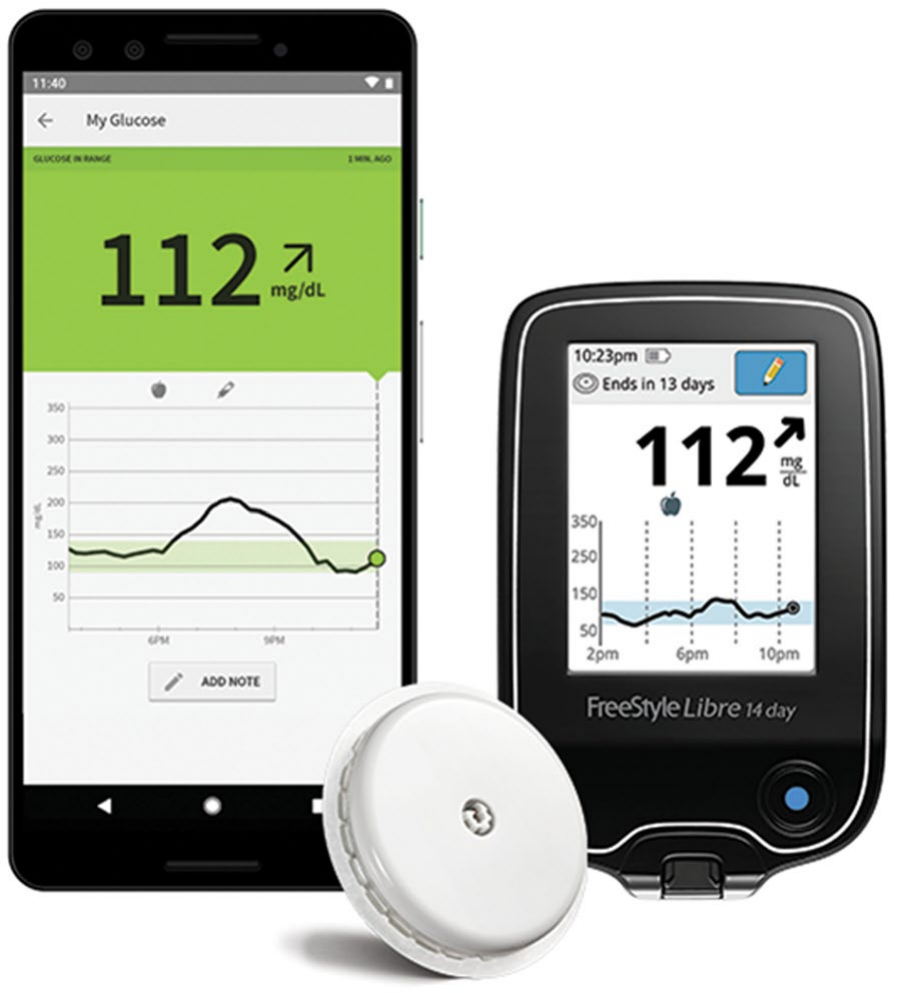
FreeStyle Libre flash glucose monitoring system (Abbott Diabetes Care, Witney, United Kingdom): sensor, reader device and its display [6]

The FreeStyle Libre™ flash glucose monitoring system (Abbott Diabetes Care, Witney, United Kingdom) consists of a sensor which is applied to the back of the upper arm and inserted below the skin [5]. The sensor measures glucose levels in the interstitial fluid every minute and stores glucose data automatically every 15 min. Each sensor lasts up to 14 days. A dedicated reader device or smartphone with near-field communication capability can be used at any time to scan the sensor to retrieve stored data. The device monitor displays the current glucose level, a trend arrow showing the direction in which glucose levels are heading, and a graph of glucose readings over the preceding 8 h (Fig. 1) [6].

The FreeStyle Libre system was approved in Europe in 2014 and, subsequently, in the United States (US) for professional use in 2016 and for personal use in 2017. The professional model uses ‘blinded’ sensors which patients bring into their physician’s office at regular intervals to have the readings downloaded. The reader is owned and maintained by the attending healthcare professional. A single reader can be used to read multiple patient sensors. The personal version uses ‘unblinded’ sensors. Patients own the reader device (or use an app on a smartphone) and can scan the sensor at any time for real-time glucose readings [7].

This review examines evidence for the flash glucose monitoring system in patients with T2D, although several real-world studies had mixed type 1 diabetes (T1D) and T2D populations.

## Search strategy

To identify clinical trials of the flash glucose monitoring system, searches were conducted of PubMed and Google Scholar from inception to 30 June 2020 using the search terms flash glucose monitoring; continuous and/or intermittent glucose monitoring; and FreeStyle Libre system. No language restrictions were applied. Reference lists of retrieved papers were hand-searched for additional clinical studies and other articles of interest. Relevant abstracts presented at the American Diabetes Association Congress in June 2020 were also considered for inclusion.

## Key evidence of flash glucose monitoring technology in type 2 diabetes

### REPLACE study

The REPLACE open-label randomized controlled trial (RCT) of adults with T2D, which compared the efficacy and safety of flash glucose monitoring (n = 149) with SMBG (n = 75), provided key supporting evidence for use of the technology in this setting [8]. The study aimed to assess the effect of flash glucose monitoring on glycemic control in patients receiving intensive insulin therapy or continuous subcutaneous insulin infusion. Although no significant difference was observed between flash technology and SMBG in the primary outcome measure of change in HbA1c at 6 months (mean − 0.29 vs. − 0.31%, respectively), prespecified subgroup analyses demonstrated several benefits (Table 1). The 6-month HbA1c level was significantly reduced in patients aged < 65 years using the flash system compared with SMBG (mean − 0.53 vs. − 0.20%; *P* = 0.030) although, for reasons not yet clear, the trend was reversed in patients aged ≥ 65 years (mean − 0.05 vs. − 0.49%; *P* = 0.008). The authors hypothesized that “the benefit for older intervention participants of being able to visualize actual or potential hypoglycemic risk prompted a more cautious approach to therapy adjustments in this vulnerable group, prioritizing hypoglycemia reduction over a more indiscriminate approach to glucose control”. Other glycemic measures significantly reduced with flash glucose monitoring compared with SMBG were time spent in hypoglycemia, frequency of hypoglycemic events and area under the concentration–time curve (AUC) for glucose, with a reduction in each of these measures in inverse proportion to the glucose level (Table 1). SMBG frequency from baseline to study end was decreased in flash glucose monitoring participants from a mean ± standard deviation (SD) of 3.8 ± 1.4 to 0.3 ± 0.7 tests/day. Treatment satisfaction, as assessed by the Diabetes Treatment Satisfaction Questionnaire, was higher in the flash glucose monitoring group compared with the SMBG group (mean ± SE 13.1 ± 0.50 vs. 9.0 ± 0.72; *P* < 0.0001). No serious adverse events (SAEs) or severe hypoglycemic events were reported in association with the device. Nine sensor adhesive reactions in six participants were described, with intensity reported as severe (n = 2), moderate (n = 6), or mild (n = 1). All reactions resolved with treatment, using mainly topical preparations.

**Table 1:** Efficacy of the flash glucose monitoring system versus self-monitoring of blood glucose in the REPLACE open-label randomized controlled trial and extension study in patients with type 2 diabetes mellitus

Randomized controlled trial	Outcome (FGMS vs. SMBG) [N = 149 vs. N = 75]		*P* value
Overall population (6 months)	Mean change from baseline in HbA1c:	− 0.29 ± 0.07 vs. − 0.31 ± 0.09%	*P* = 0.8222
Subgroup analyses (6 months)			
Age			
< 65 years	Mean change from baseline in HbA1c:	− 0.53 ± 0.09 vs. − 0.20 ± 0.12%	*P* = 0.0301
≥ 65 years		− 0.05 ± 0.10 vs. − 0.49 ± 0.13%	*P* = 0.0081
Time spent in hypoglycemia [h/day]: mean change from baseline			
Glucose < 70 mg/dL	Between-group difference:	− 43% [mean ± SE − 0.47 ± 0.13]	*P* = 0.0006
Glucose < 55 mg/dL		− 53% [− 0.22 ± 0.07]	*P* = 0.0014
Glucose < 45 mg/dL		− 64% [− 0.14 ± 0.04]	*P* = 0.0013
Frequency of hypoglycemic events [per day]: mean change from baseline			
Glucose < 70 mg/dL	Between-group difference:	− 28% [mean ± SE − 0.16 ± 0.07]	*P* = 0.0164
Glucose < 55 mg/dL		− 44% [− 0.12 ± 0.04]	*P* = 0.0017
Glucose < 45 mg/dL		− 49% [− 0.06 ± 0.02]	*P* = 0.0098
AUC [h/day × mg/dL]			
Glucose < 70 mg/dL	Between-group difference:	− 51% [mean ± SE − 7.80 ± 2.20]	*P* = 0.0005
Glucose < 55 mg/dL		− 60% [− 2.51 ± 0.76]	*P* = 0.0012
Glucose < 45 mg/dL		− 67% [− 0.70 ± 0.22]	*P* = 0.0015

Extension phase	Outcome (FGMS vs. baseline) [N = 139]		
Subgroup analyses (12 months)			
Time spent in hypoglycemia [h/day]			
Glucose < 70 mg/dL	Mean change from baseline (start of treatment phase):	− 50% [mean ± SD − 0.70 ± 1.85]	*P* = 0.0002
Glucose < 55 mg/dL		− 62% [− 0.40 ± 1.09]	*P* = 0.0002
Glucose < 45 mg/dL		− 67% [− 0.23 ± 0.73]	*P* = 0.0013
Frequency of hypoglycemic events [per day]			
Glucose < 70 mg/dL	Mean change from baseline (start of treatment phase):	− 41% [mean ± SD − 0.27 ± 0.67]	*P* < 0.0001
Glucose < 55 mg/dL		− 56% [− 0.20 ± 0.49]	*P* < 0.0001
Glucose < 45 mg/dL		− 62% [− 0.13 ± 0.35]	*P* = 0.0002
AUC [h/day × mg/dL]			
Glucose < 70 mg/dL	Mean change from baseline (start of treatment phase):	− 58% (mean ± SD − 12.73 ± 34.53]	*P* = 0.0002
Glucose < 55 mg/dL		− 65% [− 4.28 ± 12.76]	*P* = 0.0007
Glucose < 45 mg/dL		− 69% [− 1.12 ± 3.67]	*P* = 0.0021

Data from [8, 9]

BG levels are presented as mg/dL, which can be converted to mmol/L by multiplying values by 0.05551

*AUC* area under the concentration–time curve, *FGMS* flash glucose monitoring system, *HbA1c* glycosylated haemoglobin, *SMBG* self-monitoring of blood glucose

### REPLACE extension study

A total of 139 participants in the flash glucose monitoring group of the REPLACE RCT completed the 6-month treatment phase and continued into a 6-month open-access phase [9]. The mean changes from baseline (start of treatment period) in glycemic parameters measured at 12 months paralleled those measured at 6 months. Significant reductions in sensor measures of time spent in hypoglycemia, number of hypoglycemic events, and glucose AUC were observed for open-access participants at 12 months post-baseline compared with baseline, and the magnitude of change increased as glucose cut-off points decreased (Table 1).

Time in range (sensor glucose 70–180 mg/dL) remained unchanged between baseline and 12 months post-baseline (14.0 ± 4.4 vs. 14.1 ± 4.0 h). Mean ± SD frequency of SMBG decreased from 3.9 ± 1.2 tests/day at baseline to 0.2 ± 0.6 tests/day at 12 months post-baseline. During 12 months’ use of the flash glucose monitoring device there were no reports of diabetic ketoacidosis or a state of hyperosmolar hyperglycemia. As in the parent study, no SAEs were attributable to the device. Sixteen device-related adverse events (sensor adhesive or site reactions) were reported in nine participants, which were classified as severe (n = 4), moderate (n = 9) or mild (n = 3). All events resolved after treatment with mainly topical preparations.

Collectively, the 6-month REPLACE RCT and follow-on 6-month open-access study showed that, in individuals with T2D managed by intensive insulin therapy, the flash glucose monitoring system reduces hypoglycemia and is a safe alternative to SMBG. In the initial 6-month phase, the mean ± SD number of scans/day recorded by the flash glucose monitoring device was 8.3 ± 4.4 (median 6.8), which was double the frequency of blood glucose testing (median 3.8 ± 1.9 tests/day) [8]. Average sensor-scanning frequency during the extension phase was 7.1 ± 3.5 times/day (median 5.7) [9].

### Other randomized clinical trials

A pilot RCT compared the effect on glycemia of intermittent wearing of the professional flash glucose monitoring sensor with SMBG in insulin-treated T2D patients with a HbA1c level between 7.5 and 12.0% [10]. Patients performed SMBG (n = 52, control group A), or SMBG plus flash sensor worn for two 14-day periods during 4.5 months (n = 46, intervention group B), or SMBG plus flash sensor worn for four 14-day periods during 7 months (n = 50, intervention group C). No significant changes were observed within group C for sensor-derived time in range (70–180 mg/dL) from baseline to penultimate sensor wear (days 172–187; primary endpoint), with mean ± SD values of 15.0 ± 5.0 and 14.1 ± 4.7 h/day, respectively, or for the difference versus the control group at study end (days 215–230). In group C, HbA1c was reduced significantly during the study period by a mean ± SD of 0.44% ± 0.81% (*P* = 0.0003). At study end, HbA1c was significantly reduced in group C compared with the control group by an adjusted mean ± SE of 0.48% ± 0.16% (*P* = 0.004). In contrast, there was no significant difference in HbA1c between group B and control group at day 144 (*P* = 0.133). The authors concluded that flash glucose monitoring in T2D patients can “introduce clinically meaningful changes in HbA1c”.

An open-label RCT compared the effect of 10-week flash glucose monitoring (n = 53) or SMBG (n = 48) on glycemic control in patients with T2D receiving multiple daily insulin injections [11]. HbA1c was significantly reduced in the flash device group compared with SMBG, with mean changes from baseline of − 0.82% and − 0.33%, respectively (*P* = 0.005). Non-prespecified post hoc analyses showed that higher proportions of patients in the flash device group, compared with the SMBG group, had HbA1c reductions of ≥ 0.5% (68.6 vs. 30.2%; *P* < 0.001), or of ≥ 1.0% (39.2 vs. 18.6%; *P* = 0.0023). No significant differences were found in the mean ± SD perceived frequency of hypoglycemic episodes: 1.41 ± 1.29 vs. 0.75 ± 1.57, respectively (*P* = 0.066). There was a trend towards higher treatment satisfaction in the flash device group, with a mean Diabetes Treatment Satisfaction Questionnaire change version score of 2.47 ± 0.77 compared with 2.18 ± 0.83 in the standard care group (*P* = 0.053). Patients found flash glucose monitoring to be significantly more flexible than SMBG (2.28 ± 1.28 vs. 1.61 ± 1.59, *P* = 0.019), and more would recommend it to their counterparts (2.61 ± 0.86 vs. 2.19 ± 1.04, *P* = 0.023).

### Real-world observational studies

Retrospective real-world chart review studies from three European countries examined the effectiveness of flash glucose monitoring on HbA1c in adults with T2D managed by basal bolus insulin therapy [12]. Medical records from centers in Austria (n = 92), France (n = 88) and Germany (n = 183) were evaluated prior to, and following, use of the device for 90 days. Mean ± SD changes in HbA1c were − 0.9% ± 0.8% (*P* < 0.0001), − 0.8% ± 1.1% (*P* < 0.0001) and − 0.9% ± 1.1% (*P* < 0.0001), respectively. In a combined analysis of the three studies, the overall effect size was − 0.9% (*P* < 0.0001 vs. baseline). There was no significant heterogeneity between studies performed in each country (*P* = 0.711). No significant differences were recorded for changes in HbA1c according to age group, gender, body mass index, or duration of insulin use.

A real-world retrospective, observational study which analyzed data from the US electronic health record database IBM Explorys showed that de novo use of flash glucose monitoring significantly reduced HbA1c in T2D patients (n = 1084) not using bolus insulin [13]. Mean HbA1c levels decreased from 10.1% at baseline to 8.6% within 60 − 300 days of the flash glucose monitoring prescription (*P* < 0.001). Similarly, another real-world retrospective study which analyzed claims data by the Decision Resources Group, a commercial medical and pharmacy claims database, showed a significant reduction in HbA1c levels in T2D patients on long-acting insulin or non-insulin therapy after 6-month and 12-month use of flash glucose monitoring [14]. Mean HbA1c was reduced by 0.8% (from 8.5 to 7.7%) in the 6-month T2D cohort (n = 774), and by 0.6% (from 8.5 to 7.9%) in the 12-month T2D cohort (n = 207) (both *P* < 0.0001).

### Evidence interpretation

The reasons for discordance between the REPLACE trial [8] and subsequent RCTs [10, 11] and real-world studies [12–14] with respect to the effects of flash glucose monitoring on HbA1c are unknown, although the weight of evidence supports a reduction in HbA1c. It is important to highlight that patient inclusion criteria differed among studies with some patient populations using intensive insulin therapy and others not. The 12-month General Practice Optimising Structured MOnitoring To achieve Improved Clinical Outcomes (GP-OSMOTIC) trial, which compared professional-mode (masked) flash glucose monitoring with usual care (non-insulin glucose-lowering drugs, insulin, or both) in 299 adults with T2D in primary care, reported a significant reduction in mean HbA1c with flash monitoring at 6 months (− 0.5%; *P* = 0.0001) but not at 12 months (− 0.3%; *P* = 0.059), although the mean percentage of time spent in target glucose range at 12 months was 7.9% higher with flash monitoring than usual care (*P* = 0.0060) [15]. An interesting critique of the GP-OSMOTIC study, which drew attention to issues of adherence (78% at 9 months in the flash glucose monitoring group) and the absence of glucose monitoring data discussion with 43% of the intervention sample, suggested that unmasked flash glucose monitoring (i.e. a patient-based personal use system) “could be a further step from an expert-only approach to shared decision-making” [16].

Two recent real-world retrospective, observational analyses of the MarketScan database, which contains insurance billing claims for inpatient, outpatient, and pharmacy expenses, have shown benefits for flash glucose monitoring beyond glycemic control. In T2D patients not using bolus insulin (n = 7167), de novo flash glucose monitoring use (purchased between Q4 of 2017 and Q4 of 2018) significantly reduced inpatient and outpatient emergency acute diabetes events from 0.071 to 0.052 events/patient-year (hazard ratio [HR]: 0.70; 95% CI 0.57–0.85; *P* < 0.001), and all-cause hospitalization from 0.180 to 0.161 events/patient-year (HR: 0.87; 95% CI 0.78–0.98; *P* = 0.025) [17]. In T2D patients receiving fast- or short-acting insulin, flash glucose monitoring use (purchased between Q4 of 2017 and Q2 of 2018) significantly reduced acute diabetes events from 0.158 to 0.077 events/patient-year (HR: 0.49; 95% CI 0.34–0.69; *P* < 0.001) and all-cause hospitalization from 0.345 to 0.247 events/patient-year (HR: 0.72; 95% CI 0.58–0.88; *P* = 0.002) [18].

## Real-world observational studies in mixed populations of T1D and T2D

Real-world observational studies from several world regions have assessed the impact of flash glucose monitoring in often large groups of patients with T1D or T2D. The studies are described briefly and the results are presented by outcome, namely the effect of flash glucose monitoring on HbA1c, measures of hypoglycemia and hyperglycemia, and other effectiveness parameters.

A retrospective nationwide study of reimbursement claims from a French database assessed ketoacidosis rates in T1D (n = 33,203) and T2D (n = 40,955) patients who initiated flash glucose monitoring use during a 5-month study period in 2017 [19].

Four studies assessed the benefits of flash glucose monitoring mainly on HbA1c. A Dutch prospective nationwide registry study which analyzed data from 1365 participants with T1D (77.2%), T2D (16.4%), Latent Autoimmune Diabetes in Adults (4.6%) or maturity-onset diabetes of the young (0.5%) examined the effect of flash glucose monitoring on HbA1c, disease burden and well-being [12]. A cohort study using data from the Swedish National Diabetes Register (January 2014–June 2019) assessed the effectiveness of the FreeStyle Libre system on HbA1c reduction [21]. A meta-analysis of 29 clinical trials and real-world studies, of which 25 reported longitudinal HbA1c data in 1723 participants with T1D or T2D using the FreeStyle Libre system, examined the impact of flash glucose monitoring on HbA1c [22] A small study from Israel assessed the impact of flash glucose monitoring on HbA1c in T2D (n = 25) and T1D (n = 6) patients [23].

Other studies assessed the impact of increased scanning frequency on glycemic measures. A real-world European analysis examined deidentified data from more than 50,000 users worldwide of the FreeStyle Libre system who had performed more than 60 million scans over a 20-month period [24]. To assess the role of flash glucose monitoring in early and late changes of glycemic markers under real-life conditions, a longitudinal study analyzed deidentified glucose results from 6802 flash monitors after stratification into high, medium and low-risk groups based on tertiles of time spent in hypoglycemia (min/day < 70 mg/dL) or hyperglycemia (h/day > 240 mg/dL) [25]. Another large real-world study analyzed deidentified glucose and user scanning data (250 million glucose readings, 37.1 million glucose scans) collected over a 4-year period from Spanish users (n = 22,949) to determine the relationship between testing frequency and glycemic parameters [26]. An interesting study from Brazil analyzed glucose results captured from launch of the FreeStyle Libre flash glucose monitor in 2016 and compared them with global population data collected between September 2014 and December 2018 [27]. Data were analyzed from 688,640 readers and 7,329,052 sensors worldwide, including 17,691 readers and 147,166 sensors from Brazil.

### Effect on HbA1c

Four studies showed that flash glucose monitoring improved glycemic control, as assessed by HbA1c, compared with prior to its use (Table 2) [20–23]. In the Dutch prospective registry study, estimated HbA1c decreased from 8.0% before use of flash glucose monitoring to 7.6% after 6 months of use (*P* < 0.001) and remained steady at 7.6% at 12 months (*P* < 0.001). The 12-month difference in estimated HbA1c was more pronounced in patients with T2D (n = 223) than T1D (n = 1054) [20]. Swedish National Diabetes Register data also showed a significant decrease in HbA1c (method of measurement unspecified) before and after incident FreeStyle Libre use, with a mean change of − 0.33% for T1D patients (n = 8,316) and − 0.52% for T2D patients (n = 538) at 12 months (both *P* < 0.0001) [21]. The meta-analysis of clinical trials and real-world studies of flash glucose monitoring indicated a mean change in laboratory HbA1c of − 0.55% at 2–4 months, with a negligible difference (− 0.56% and − 0.54%, respectively) observed between adults (n = 1023) and children and adolescents (n = 447) [22]. Longitudinal analysis of studies involving adult subjects (n = 1276) showed that laboratory HbA1c was reduced within the first 2 months of use, and that changes were sustained for up to 12 months [22], thus confirming a trend observed in a previous small study of flash glucose monitoring in patients with HbA1c ≥ 7.5%, in which the majority of change from baseline in mean HbA1c (method of measurement unspecified) occurred by 8 weeks (− 1.33%; *P* < 0.0001) and was maintained at 24 weeks (− 1.21%; *P* = 0.009) [23].

**Table 2:** Effect of flash glucose monitoring use and scanning frequency on glycosylated hemoglobin (HBA1c) levels in real-world studies of patients with type 1 and type 2 diabetes

Study (population)	Effect of:	HbA1c (%)
Fokkert et al. [20] T1D, n = 1054; T2D, n = 223; Other, n = 88	Before vs. after FGMS use on estimated HbA1c	At baseline: 8.0% (95% CI 7.9–8.1) At 6 months: 7.6% (95% CI 7.5–7.7); *P* < 0.001 *vs*. baseline At 12 months: 7.6% (95% CI 7.6–7.7); *P* < 0.001 *vs*. baseline
Eeg-Olofsson et al. [21] T1D, n = 8316; T2D, n = 538	Before vs. after FGMS use on HbA1c (method of measurement not specified)	T1D: 8.1% at baseline. Mean change –0.33% (95% CI − 0.36 to − 0.31); *P* < 0.0001 T2D: 8.6% at baseline. Mean change –0.52% (95% CI − 0.63 to − 0.40); *P* < 0.0001
Evans et al. [22] Meta-analysis of 29 studies; n = 1723 with T1D or T2D	FGMS use on laboratory HbA1c	In adults at 2–4 months: mean change − 0.56% (95% CI − 0.76 to − 0.36) In children and adolescents at 2–4 months: mean change − 0.54% (95% CI − 0.84 to − 0.23)
Ish-Shalom et al. [23] T1D, n = 6; T2D, n = 25	FGMS use on HbA1c (method of measurement not specified)	In patients with HbA1c ≥ 7.5% At 8 weeks: mean change − 1.33 ± 0.29%; *P* < 0.0001 At 24 weeks: mean change − 1.21 ± 0.42%; *P* = 0.009
Dunn et al. [24] n > 50,000	↑ Scanning frequency on estimated HbA1c	Highest (48.1 scans/day) vs. lowest (4.4 scans/day) scan rate group: 6.7% (95% CI 6.7–6.8) vs. 8.0% (95% CI 7.9–8.0; *P* < 0.001
Gomez-Peralta et al. [26] n = 22,949	↑ Scanning frequency on estimated HbA1c	Highest (39.6 scans/day) vs. lowest (3.9 scans/day) scan rate group: 6.9% (95% CI 6.9–7.0) vs. 8.0% (95% CI 8.0–8.1); *P* < 0.001
Calliari et al. [27] Brazil: 17,691 readers and 147,166 sensors Worldwide: 688,640 readers and 7,329,052 sensors	↑ Scanning frequency on estimated HbA1c	Brazil: Highest (43.1 scans/day) *vs*. lowest (3.6 scans/day) scan rate group: 6.7% (95% CI 6.6–6.8) vs. 7.6% (95% CI 7.4–7.7); *P* < 0.01 Worldwide: Highest (37.8 scans/day) *vs*. lowest (3.4 scans/day) scan rate group: 6.7% (95% CI 6.7–6.7) vs. 8.1% (95% CI 8.1–8.2); *P* < 0.01

BG levels are presented as mg/dL, which can be converted to mmol/L by multiplying values by 0.05551

*BG* blood glucose, *FGMS* flash glucose monitoring system, *HbA1c* glycosylated haemoglobin, *T1D* type 1 diabetes, *T2D* type 2 diabetes

↑ indicates increased

Additional studies showed that people who scan more frequently tend to have lower HbA1c (Table 2) [24, 26, 27]. In the European real-world analysis, greater scanning frequency from 4.4 (lowest) to 48.1 (highest) scans/day was associated with a reduction in estimated HbA1c from 8.0% to 6.7% (*P* < 0.001) [24]. In the real-world study of Spanish users of the flash glucose monitoring device, estimated HbA1c was significantly lower in the highest (39.6 scans/day) versus lowest (3.9 scans/day) scan frequency group (6.9 vs. 8.0%; *P* < 0.001) [26]. Similarly, the Brazilian study found that, in line with worldwide data, increased scanning frequency in Brazil was associated with better glycemic control, as evidenced by a lower estimated HbA1c in the highest (43.1 scans/day) versus lowest (3.6 scans/day) scan rate groups (6.7 vs. 7.6%; *P* < 0.01) [27].

### Effect on measures of hypoglycemia and hyperglycemia

Results from four real-world studies showed that increased scanning frequency of the flash monitoring device was associated with benefits on glycemic measures apart from HbA1c (Table 3) [24–27].

**Table 3:** Effect of flash glucose monitoring scanning frequency on measures of hypoglycemia and hyperglycemia in real-world studies of patients with type 1 and type diabetes

Study	Effect of	Time spent in hypoglycemia	Time spent in hyperglycemia	Time in range
Dunn et al. [24] n > 50,000	↑ Scanning frequency: 48.1 highest and 4.4 lowest scans/day (mean 16.3 scans/day)	Highest vs. lowest scan rate group: BG < 70 mg/dL: ↓ 15%; 79.3 vs. 93.4 min/day; *P* < 0.001 BG < 56 mg/dL: ↓ 40%; 26.2 vs. 43.4 min/day; *P* < 0.001 BG < 45 mg/dL: ↓ 49%; 11.9 vs. 23.4 min/day; *P* < 0.001	Highest vs. lowest scan rate group: BG > 180 mg/dL: ↓ 44%; 5.9 vs. 10.5 h/day; *P* < 0.001	Highest vs. lowest scan rate group: BG 70–180 mg/dL: ↑ 40%; 16.8 vs. 12.0 h/day; *P* < 0.001
Jangam et al. [25] Hypoglycemia n = 2,268 or hyperglycemia n = 2,268	Comparison between first and last 14-day periods of sensor wear^a^, after stratification of results based on risk of hypoglycemia or hyperglycemia and scanning frequency^b^	High-risk hypoglycemia group (BG ≤ 70 mg/dL): ↓ 19.5% from 200 ± 3 to 161 ± 5 min/day in higher-frequency scanners (mean 20.3 scans/day); *P* < 0.0001 ↓ 24.5% from 196 ± 3 to 148 ± 4 min/day in medium-frequency scanners (mean 11.6 scans/day); *P* < 0.0001 ↓ 24.5% from 204 ± 3 to 154 ± 4 min/day in low-frequency scanners (mean 7 scans/day); *P* < 0.0001	High-risk hyperglycemia group (BG > 240 mg/dL): ↓ 14.2% from 5.7 ± 0.10 to 4.9 ± 0.14 h/day in higher-frequency scanners (mean 18.1 scans/day); *P* < 0.0001 ↓ 6.3% from 5.8 ± 0.09 to 5.5 ± 0.13 h/day in medium-frequency scanners (mean 10.5 scans/day); *P* = 0.02 No effect in low-frequency scanners (mean 6.2 scans/day)	
Gomez-Peralta et al. 2020 [26] n = 22,949	↑ Scanning frequency: 39.6 highest and 3.9 lowest scans/day (mean 13 scans/day)	Highest vs. lowest scan rate group: BG < 70 mg/dL: ↓ 14%; 85.3 (95% CI 79.3–91.2) vs. 99.2 (95% CI 93.9–104.4) min/day; *P* < 0.001 BG < 54 mg/dL: ↓ 37%; 29.7 (95% CI 26.6–32.8) vs. 46.8 (95% CI 43.6–49.9) min/day; *P* < 0.001	Highest vs. lowest scan rate group: BG > 180 mg/dL: ↓ 37%; 6.9 (95% CI 6.7–7.2) vs. 10.9 (95% CI 10.6–11.2) h/day; *P* < 0.001	Highest vs. lowest scan rate group: BG 70–180 mg/dL: ↑ 36%; 15.6 (95% CI 15.4–15.9) vs. 11.5 (95% CI 11.2–11.7) h/day; *P* < 0.001
Calliari et al. [27] Brazil: 17,691 readers and 147,166 sensors Worldwide: 688,640 readers and 7,329,052 sensors	↑ Scanning frequency Brazil: 43.1 highest and 3.6 lowest scans/day (average 14 scans/day) Worldwide: 37.8 highest and 3.4 lowest scans/day (average 12 scans/day)	Highest *vs*. lowest scan rate group (BG < 54 mg/dL): Brazil: 27.1 (95% CI 23.8–30.5) vs. 28.3 (95% CI 25.0–31.5) min/day; *P* = 0.64 Worldwide: 22.9 (95% CI 22.5–23.4) vs. 31.1 (95% CI 30.6–31.6) min/day; *P* < 0.01	Highest *vs*. lowest scan rate group (BG > 180 mg/dL): Brazil: 6.0 (95% CI 5.7–6.3) vs. 8.7 (95% CI 8.3–9.1) h/day; *P* < 0.01 Worldwide: 5.8 (95% CI 5.8–5.9) vs. 10.8 (95% CI 10.7–10.8) h/day; *P* < 0.01	Highest *vs*. lowest scan rate group (BG 70–180 mg/dL): Brazil: 16.6 vs. 14.2 h/day; *P* < 0.01 Worldwide: 17.0 vs. 12.1 h/day; *P* < 0.01

BG levels are presented as mg/dL, which can be converted to mmol/L by multiplying values by 0.05551

*BG* blood glucose, *CI* confidence interval

↑ indicates increased; ↓ indicates decreased/reduced

^a^Scanning frequency decreased gradually from > 18 scans/day during first sensor use to ≈ 15 scans/day at 2 months, and was maintained at the lower level for the remainder of the 6-month analysis period

^b^Glucose results were analyzed after being divided into high, medium and low-risk groups based on tertiles of time spent in hypoglycemia (min/day < 70 mg/dL) or hyperglycemia (h/day > 240 mg/dL), and further subdivision into tertiles of glucose scanning frequency (high, medium, low)

In the European analysis, greater scanning frequency was inversely correlated with time spent in hypoglycemia and hyperglycemia. For blood glucose levels < 70 mg/dL, < 56 mg/dL and < 45 mg/dL, time in hypoglycemia was lower by 15%, 40% and 49%, respectively (all *P* < 0.001) in the highest (48.1 scans/day) compared with the lowest (4.4 scans/day) scan rate group. Highest versus lowest scanning frequency was also associated with a 44% decrease (*P* < 0.001) in time spent in hyperglycemia and a 40% increase in time in range [24]. Six-month data from the real-world longitudinal study showed that, in the high-risk hypoglycemia group, flash glucose monitoring significantly (*P* < 0.0001) reduced the mean time spent in hypoglycemia (blood glucose ≤ 70 mg/dL) from the first to last 14-day periods of the study, irrespective of scanning frequency (high, medium, or low). In the high-risk hyperglycemia group, flash glucose monitoring reduced the time spent in hyperglycemia (blood glucose > 240 mg/dL) by 0.8 h/day in higher-frequency scanners (*P* < 0.0001), by 0.3 h/day in medium-frequency scanners (*P* = 0.02), and had no effect in low-frequency scanners from the first to last 14-day periods of the study [25].

In the real-world study of Spanish users of the flash glucose monitoring device, glucose parameters progressively improved as average scanning frequency increased from the lowest (3.9 scans/day) to highest (39.6 scans/day) scan rate group. Time in hypoglycemia for blood glucose thresholds of < 70 mg/dL and ≤ 54 mg/dL, respectively, was decreased by 14% and 37% in the highest versus lowest scan rate group. Respective times in hypoglycemia for the highest and lowest scan rate groups were 85.3 and 99.2 min/day (*P* < 0.001) for blood glucose < 70 mg/dL; and 29.7 min/day and 46.8 min/day for blood glucose ≤ 54 mg/dL. Time spent in hyperglycemia (blood glucose > 180 mg/dL) was decreased by 37% (*P* < 0.001), and time in range was increased by 36% (*P* < 0.001) and in the highest versus lowest scan rate group [26]. A comparison of sensor data derived from flash glucose monitoring users in Brazil and worldwide showed significant (*P* < 0.01) improvements in time spent in hyperglycemia (blood glucose > 180 mg/dL) associated with highest versus lowest scanning frequency: 43.1 and 3.6 scans/day, respectively, in Brazil; 37.8 and 3.4 scans/day, respectively, worldwide [27]. In both populations, greater scanning frequency also increased time in range (blood glucose 70–180 mg/dL) [27], a glycemic metric gaining international recognition as a useful and appropriate clinical target [28].

### Other effects

The retrospective study analyzing reimbursement claims from a French database showed a marked reduction in ketoacidosis rates in patients who initiated flash glucose monitoring during a 5-month study period in 2017. The hospitalization rate for ketoacidosis (excluding incidence for coma) was reduced by 52% (from 5.5 to 2.6 per 100 patient-years) and by 47% (from 1.7 to 0.9 per 100 patient-years) in T1D and T2D patients, respectively [19].

In the Dutch prospective registry study, 12-month use of flash glucose monitoring significantly reduced the proportion of patients experiencing any hypoglycemic event from 93.5 to 91.0%; the proportion of diabetes-related hospitalization from 13.7 to 4.7%; and work absenteeism from 18.5 to 7.7% (all comparisons *P* < 0.05). In addition, flash glucose monitoring improved 12-month well-being scores, with changes from baseline of 0.03 (95% CI 0.01–0.05) in the EuroQol 5D tariff, 4.4 (95% CI 2.1–6.7) in the EQ-visual analogue scale, and 3.3 (95% CI 2.1–4.4) in the 12-Item Short Form Health Survey v2 mental component score [20].

## Conclusion

Data on use of flash glucose monitoring in people with T2D are accumulating steadily. Although no significant changes in HbA1c levels were observed in the REPLACE trial which compared flash glucose monitoring with SMBG in adults with T2D receiving intensive insulin therapy, additional RCTs and real-world chart review studies have since documented that flash glucose monitoring significantly reduces HbA1c from baseline. Real-world studies of both populations of patients with T1D or T2D indicate that flash glucose monitoring is associated with less time spent in hypoglycemia or hyperglycemia, and greater time in target glucose range. Higher scanning frequency was associated with better glycemic metrics, particularly among patients at higher risk of hyperglycemia or hypoglycemia who spent significantly less time above or below target values. Other benefits reported with use of flash glucose monitoring include reductions in acute diabetes events and all-cause hospitalizations, reductions in hospitalized ketoacidosis episodes (except comas), improved well-being and decreased disease burden, and greater treatment satisfaction. Taken together, the evidence indicates that flash glucose monitoring is suitable and safe for use in T2D patients, especially those who could benefit from tighter glycemic control and associated reduction in disease burden.

## Data Availability

Data sharing is not applicable since it is a review article.
